# Emotional and tangible social support in a German population-based sample: Development and validation of the Brief Social Support Scale (BS6)

**DOI:** 10.1371/journal.pone.0186516

**Published:** 2017-10-12

**Authors:** Manfred E. Beutel, Elmar Brähler, Jörg Wiltink, Matthias Michal, Eva M. Klein, Claus Jünger, Philipp S. Wild, Thomas Münzel, Maria Blettner, Karl Lackner, Stefan Nickels, Ana N. Tibubos

**Affiliations:** 1 Department of Psychosomatic Medicine and Psychotherapy, University Medical Center Mainz, Mainz, Germany; 2 Medical Clinic for Cardiology, Angiology and Intensive Care Medicine, University Medical Center Mainz, Mainz, Germany; 3 Preventive Cardiology and Preventive Medicine, Department of Medicine 2, University Medical Center Mainz, Mainz, Germany; 4 Center for Thrombosis and Hemostasis, University Medical Center Mainz, Mainz, Germany; 5 German Center for Cardiovascular Research (DZHK), partner site Rhine Main, University Medical Center Mainz, Mainz, Germany; 6 Institute for Medical Biostatistics, Epidemiology and Informatics (IMBEI), University Medical Center Mainz, Mainz, Germany; 7 Institute for Clinical Chemistry and Laboratory Medicine, University Medical Center Mainz, Mainz, Germany; 8 Department of Ophthalmology University Medical Center Mainz, Mainz, Germany; University Medical Center Hamburg-Eppendorf, GERMANY

## Abstract

Aim of the study was the development and validation of the psychometric properties of a six-item bi-factorial instrument for the assessment of social support (emotional and tangible support) with a population-based sample. A cross-sectional data set of *N* = 15,010 participants enrolled in the Gutenberg Health Study (GHS) in 2007–2012 was divided in two sub-samples. The GHS is a population-based, prospective, observational single-center cohort study in the Rhein-Main-Region in western Mid-Germany. The first sub-sample was used for scale development by performing an exploratory factor analysis. In order to test construct validity, confirmatory factor analyses were run to compare the extracted bi-factorial model with the one-factor solution. Reliability of the scales was indicated by calculating internal consistency. External validity was tested by investigating demographic characteristics health behavior, and distress using analysis of variance, Spearman and Pearson correlation analysis, and logistic regression analysis. Based on an exploratory factor analysis, a set of six items was extracted representing two independent factors. The two-factor structure of the Brief Social Support Scale (BS6) was confirmed by the results of the confirmatory factor analyses. Fit indices of the bi-factorial model were good and better compared to the one-factor solution. External validity was demonstrated for the BS6. The BS6 is a reliable and valid short scale that can be applied in social surveys due to its brevity to assess emotional and practical dimensions of social support.

## Introduction

The presence of social relationships has been associated with a great number of mental and physical health outcomes. Social ties have a beneficial influence on the maintenance of psychological well-being and health-promoting behavior [1]. Structural features of social integration comprise of characteristics such as marital status, living alone or the size of a person’s social network. Functional parameters usually refer to the perceived, respectively anticipated support from the social environment [2, 3]. Loneliness refers to a discrepancy between desired and available social relationships [4], a perceived sense of isolation [5], separateness or exclusion [6].

Following the seminal work of House et al. [7], a recent meta-analysis of 148 studies (totaling up to over 308.000 participants) found a 50% increased likelihood of survival in participants with stronger social ties taking into account initial health status, cause of death, and duration of follow-up. This was true for social integration and for social support, respectively its lack in loneliness [8]. Another meta-analysis [9] found that low social participation, less frequent social contact and more loneliness, but not size of the social network were statistically associated with incident dementia with similar rates of likelihood.

There are two models explaining the association of social relationships to health outcomes. The main effects model proposes beneficial cognitive, emotional, behavioral and biological influences of social relationships, such as providing stimulation and activity, stimulating brain activity, modeling health behavior, or promoting self-esteem or purpose in life by providing meaningful social roles. The buffering hypothesis proposes that social relationships provide emotional, informational or tangible resources that promote adaptation in case of stress or adversity [10, 11].

Many scales have been proposed to assess social support in the last decades, e.g. Duke-UNC Functional Social Support Questionnaire [12], Oslo Social Support Scale [13], Sarason’s Social Support Questionnaire [14], Interpersonal Support Evaluation List [15]. One established scale for measuring social support is the MOS (Medical Outcomes Study) Social Support Survey, which was developed based on responses of 2,987 chronically ill adults [16]. Of the 19 original items, one was dropped as it belonged to two scales. 18 items assess four dimensions of support: emotional-informational (emotional support and guidance or advice), tangible support (i.e. material aid or assistance), affectionate support (expressing love and affection), and positive social interaction (provided by individuals with whom it is fun to do things). A number of subsequent studies have also been mostly done on medically ill patients, e.g. American breast cancer patients [17] or Spanish outpatients from a primary care center [18]. Given its easy comprehensibility with a single sentence stem “If you needed it, how often is someone available…”, the instrument lends itself to use in a wide range of populations and age groups. Except for the Australian study of Holden et al. [19], population-based studies, however, have been scarce. One reason limiting its use may be the length of the scale. So far, a number of different scales have been developed, based on the initial item pool of the MOS trial with a range from 4 [20], 6 [19], 8 [17] and 12-item scales [20]. The numbers of factors also varies from 1 [18], 2 [17] to 4 [20]. Yet, even those papers endorsing more than one dimension usually used the total score of the respective scale for validation (e.g. [17, 19]), and no evidence has been presented for differential significance of the respective subscales.

Social support has been found to improve mental health and health-promoting behavior [1]. Most analyses identified the two dimensions of emotional-informational and tangible support [19–21]. There is some evidence that the distinction of both facets, emotional-informational and tangible support, are crucial [1, 22, 23], as affective support and material assistance can be provided by different environmental resources. Emotional-informational support mainly covers psychological components, particularly emotional and cognitive needs. Tangible support on the other hand, is based on practical support, such as material aid and behavioral assistance [16]. So far, an instrument accounting for the need of factorial differentiation of these two facets of social support is lacking. Especially the development of a bi-factorial questionnaire which can be used in population-based studies to collect representative data seems reasonable.

For this reason, the aim of this paper was to develop and validate a Brief Social Support Scale (BS6) based on the MOS Social Support Survey that reliably assesses emotional-informational as well as tangible support. The database was provided by the Gutenberg Health Study (GHS) which is a German population-based, prospective, observational single-center cohort study. To the best of our knowledge, this study is the first validating items of the MOS Social Support Survey including country specific data based on representative data collection. A factor-analytical approach was chosen to extract a bi-factorial brief version of the MOS Social Support Survey. In order to indicate external validity of the scales, socio-demographic data and health-related variables were analyzed which have been associated with facets of social-support [17, 24].

The following hypotheses were tested:

1. There are two underlying independent factors representing aspects of social support, namely emotional-informational support and tangible support.
2. Emotional-informational support and tangible support are associated with distress (depression, generalized anxiety, social phobia, panic disorder, suicidal ideation), physical and mental well-being, and health behavior (smoking, BMI, physical activity, alcohol consumption).

## Methods

### Study sample

We investigated cross-sectional data of *N* = 15,010 participants enrolled in GHS from April 2007 to April 2012 [25]. The GHS is a population-based, prospective, observational single-center cohort study in the Rhein-Main-Region in western Mid-Germany. The study protocol and study documents were approved by the local ethics committee of the Medical Chamber of Rhineland-Palatinate, Germany (reference no. 837.020.07; original vote: 22.3.2007, latest update: 20.10.2015). The primary aim of the study is to evaluate and improve cardiovascular risk stratification. The sample was drawn randomly from the local registry in the city of Mainz and the district of Mainz-Bingen. A multi-method approach was used for recruitment (see also [25]). For this purpose, an algorithm was developed defining time frames for establishing contact with the study participants. Overall, participants were contacted up to three times by postal means and up to 20 times by telephone. Participants who did not participate in the GHS study were asked to take part in a structured interview in order to gain information for non-responder analyses with regard to selection bias. The sample was stratified 1:1 for sex and residence and in equal strata for decades of age. Inclusion criteria were age 35 to 74 years and written informed consent. Persons with insufficient knowledge of German language, or physical and mental inability to participate were excluded. Based on the interim analysis 5.8% were excluded because of the exclusion criteria. The response rate (defined as the recruitment efficacy proportion, i.e. the number of persons with participation in or appointment for the baseline examination divided by the sum of number of persons with participation in or appointment for the baseline examination plus those with refusal and those who were not contactable) was 60.3%. Sociodemographic details of the sample are displayed in S1 Table.

### Materials and assessment

The 5-hour baseline-examination in the study center comprised evaluation of prevalent classical cardiovascular risk factors and clinical variables, a computer-assisted personal interview, laboratory examinations from a venous blood sample, blood pressure and anthropometric measurements. In general, all examinations were performed according to standard operating procedures by certified medical technical assistants.

### Questionnaires

The MOS Social Support Survey with 20 items was devised to assess social support based on the four dimensions of emotional-informational, tangible, affectionate support und positive social interaction. Based on samples of patients with medical diseases, Moser et al. [17] devised a brief scale with 8 items and two subscales (emotional and instrumental support, 4 items, each). GHS participants filled in part of the MOS assessing emotional-informational and tangible support (9 items). Items are rated from 1 = never, 2 = occasionally, 3 = mostly to 4 = always available. An additional item was assessed to estimate the number of persons the participants can count on in cases of emergency (no one to 3 and more).

Depression was measured by the Patient Health Questionnaire (PHQ-9), which quantifies the frequency of being bothered by each of the 9 diagnostic criteria of Major Depression over the past 2 weeks. Responses are summed to create a score between 0 and 27 points. A PHQ-9 sum score of ≥ 10 was used for the definition of caseness for depression yielding a sensitivity of 81% and a specificity of 82% for any depressive disorder [26].

Generalized anxiety was assessed with the two screening items of the short form of the GAD-7 (Generalized Anxiety Disorder [GAD]-7 Scale [27]). “Feeling nervous, anxious or on edge” and “Not being able to stop or control worrying”. Subjects can answer these items with 0 = “not at all”, 1 = “several days”, 2 = “over half the days”, and 3 = “nearly every day”. A sum score of 3 and more (range 0–6) out of these two items indicates generalized anxiety with good sensitivity (86%) and specificity (83%). Both the GAD-7 and its two core items (GAD-2) have been shown to perform well as a screening tool for all anxiety disorders [28].

### Computer-assisted personal interview

During the computer-assisted personal interview participants were asked whether they had ever received the definite diagnosis of any depressive disorder (medical history of lifetime diagnosis of any depressive disorder, MH of Depression) by a physician. The socioeconomic status (SES) was defined according to Lampert’s and Kroll’s scores of SES with a range from 3 to 27 (3 indicates the lowest SES and 27 the highest SES) [29].

### Statistical analysis

For the scale development, a factor-analytical approach was used. Item selection was based on exploratory factor analysis. Subsequently, a confirmatory factor analysis for testing the postulated bi-factorial model was conducted.

Descriptive analyses, correlation and regression analyses were performed to validate the subscales with external criteria. Variables were reported as absolute numbers, percentage or mean with standard deviation or medians with 25th and 75th percentiles as appropriate. For comparison of groups Cochran-Armitage Test or Jonckheere-Terpstra Test were performed. All reported p-values correspond to 2-tailed tests. As this part of the study is explorative, no adjustments for multiple testing have been done. P-values are given for descriptive reasons only. Due to the large number of tests, p-values should be interpreted with caution and in connection with effect estimates. Statistical analyses were performed using SAS for Windows 9.4 TS Level 1M1 (SAS Institute Inc.) Cary, NC, USA. For factor analyses, the software R 3.2.3 (package lavaan 0.5–22) was used.

## Results

### Factor analyses: Exploratory factor analysis and confirmatory factor analysis

**Fig 1:**
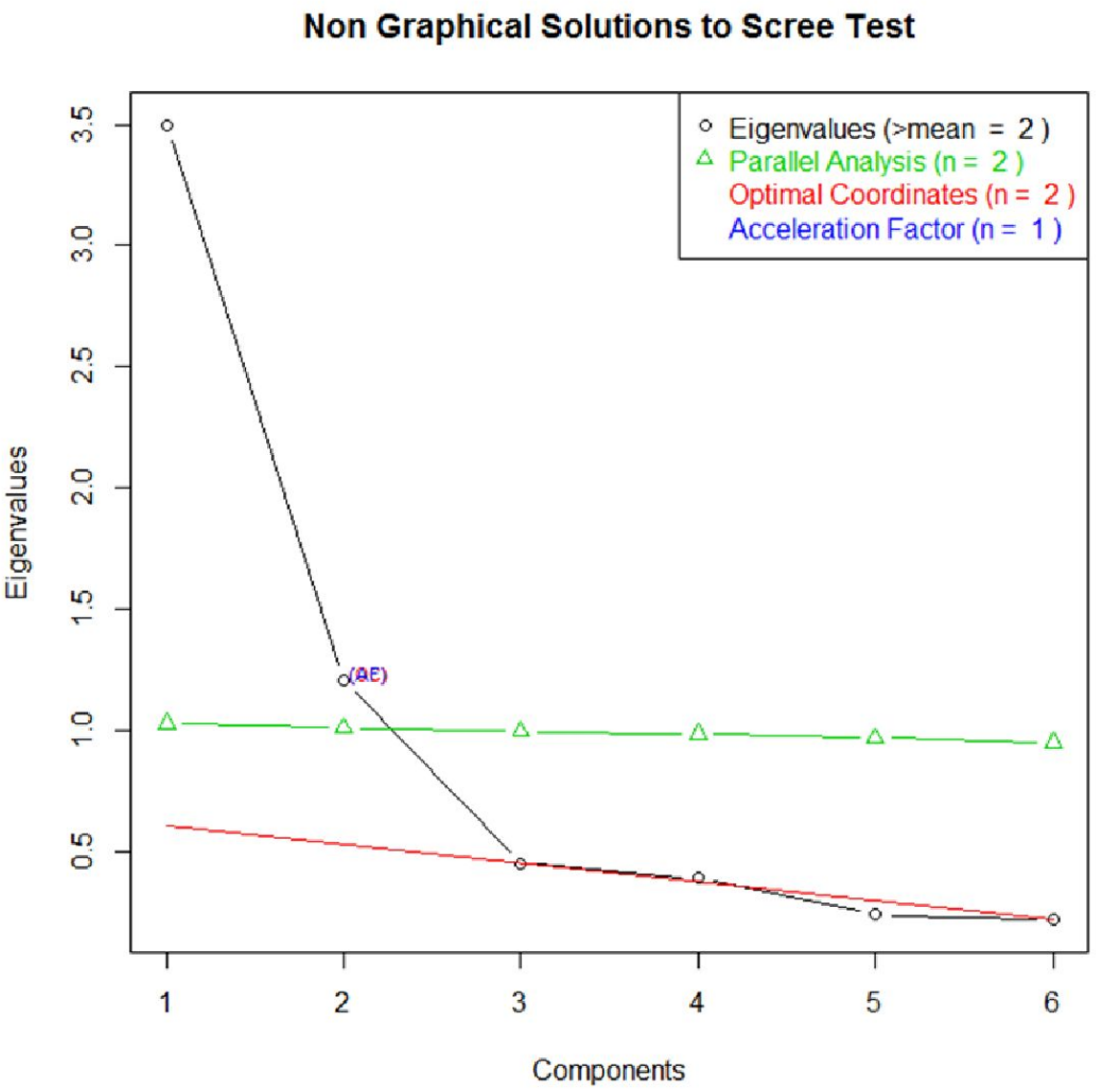
Factor extraction based on exploratory factor analysis.

Two sets of factor analyses were conducted after splitting the sample in two subsamples by randomization: An exploratory factor analysis with varimax rotation (*n* = 7,256) and a confirmatory factor analysis (*n* = 7,255). Table 1 and Fig 1 present the findings from the exploratory factor analysis with varimax rotation. As we were interested in developing a brief scale, we deleted three items with overlapping factor loadings (“to do something enjoyable with 38/.44“, “to have a good time with .56/.46”, “whose advice you really want .65/.39”). Based on 6 items (see Table 1), we identified two independent factors (r = .07) by means of exploratory factor analysis and referring to the scree-test (see Fig 1). Factor 1 explained 35% and factor 2 explained 34% of the variance. The first factor comprises items representing emotional-informational support, the second factor tangible support.

**Table 1:** Item statistics including mean score, standard deviation, factor loadings, and corrected item-total-correlation.

English	German	Mean (±SD)	Factor loadings		r_it_	r_it_
			Factor 1* (emotional-informational support)	Factor 2* (tangible support)	Factor 1*	Factor 2*
If you needed it, how often is someone available…	Wie häufig steht Ihnen folgende Unterstützung durch andere Menschen zur Verfügung?					
*Tangible support*						
To take you to the doctor if you need it	Jemand, der Sie zum Arzt fährt, wenn es nötig ist	3.5 (±0.8)		0.64	0.30	0.61
To prepare your meals if you are unable to do it yourself	Jemand, der Ihnen Essen zubereitet, wenn Sie dazu nicht in der Lage sind	3.5(±0.8)		0.90	0.27	0.78
To help with daily chores if you were sick	Jemand, der Ihnen bei alltäglichen Arbeiten hilft, wenn Sie krank sind	3.4(±0.8)		0.80	0.30	0.75
*Emotional-informational support*						
To give you good advice about a crisis	Jemand, der Ihnen in schwierigen Situationen gute Ratschläge gibt	3.4(±0.8)	0.69		0.64	0.33
To confide in or talk to about yourself or your problems	Jemand, dem Sie sich anvertrauen oder mit dem Sie über persönliche Probleme sprechen können	3.4(±0.8)	0.87		0.77	0.28
Who understands your problems	Jemand, der Ihre Probleme versteht	3.3(±0.8)	0.82		0.76	0.28

* Calculated by exploratory factor analysis.

**Fig 2:**
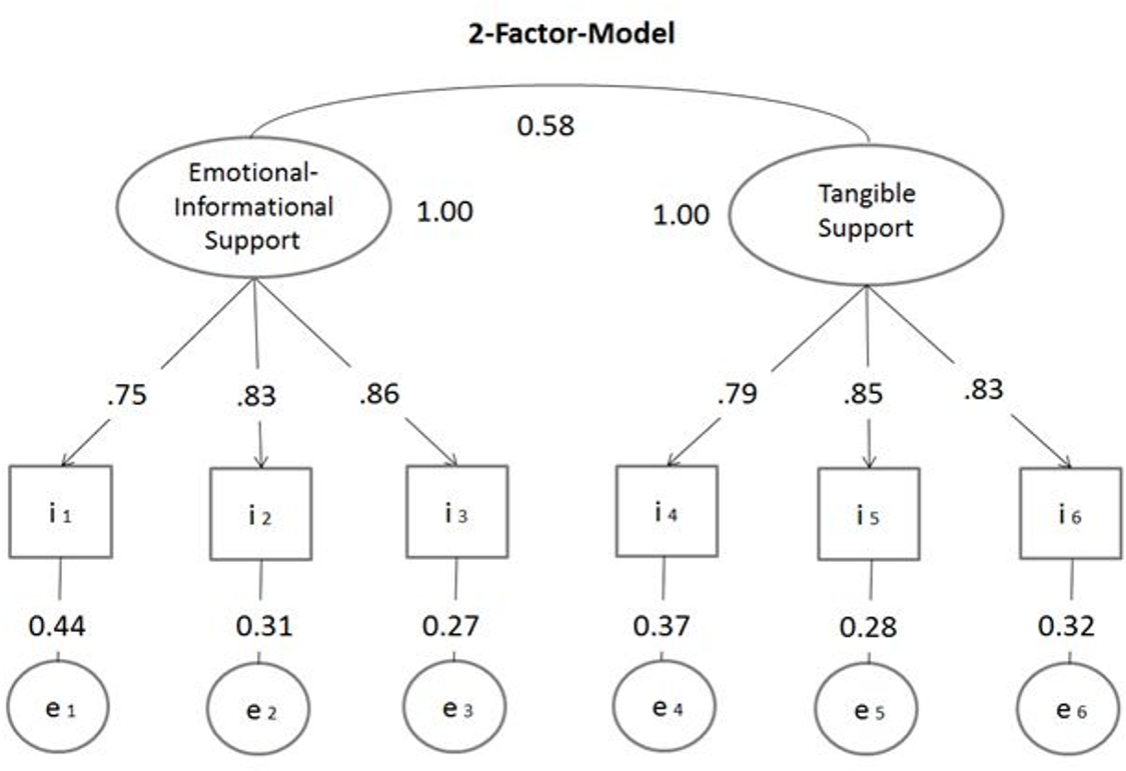
Factor structure of the Brief Social Support Scale (BS6). *Note*: Confirmatory factor analysis with completely standardized solution.

Reliability of the subscales indicated by Cronbach´s alpha was satisfactory: emotional-informational support *α* = .87, tangible support *α* = .86, overall *α* = .86. Model fit was evaluated by using following model fit indices: Chi-square statistic; the comparative-fit-index (CFI) and the Tucker-Lewis Index (TLI) to describe incremental fit; the root mean square error of approximation (RMSEA) was used as an absolute measure of fit. Values of TLI and CFI close to .95 or higher indicate a better fit. RMSEA should be 0.08 or smaller [30]. Confirmatory factor analyses yielded a good fit for the bi-factor-model (see Fig 2): RMSEA .030 (90% CI .023-.038), CFI = .996, TLI = .993, and SRMR = .029. In contrast, the one-factor-model reached an unacceptable fit: RMSEA .127 (90% CI .121-.134), CFI = .929, TLI = .882, and SRMR = .125.

### Association of social support and its subscales with demographic characteristics health behavior, and distress

Table 2 gives an overview according to different degrees of social support. Sum scores of social support were stratified by quartiles into low (6–11), moderate (at least occasional support), high (at least mostly supported) to very high (maximum count, always supported).

**Table 2:** Social support and its associations with demographic characteristics, health behavior, and distress.

	Social support descriptive statistics					Correlation coefficients Pearson/ Spearman		
	low 6–11 N = 444 (3.0%)	moderate 12–17 N = 2213 (15.2%)	high 18–23 N = 8032 (55.2%)	very high 24 N = 3873 (26.6%)	p-value	Social support	Emotional-informational support	Tangible support
Age (in years, M, SD)	56.9 (11.1)	55.1 (10.9)	54.4 (11.0)	55.6 (11.3)	.15	-0.01	0.01/ 0.04	-0.03/ -0.03
Women (%)	53.6	54.1	48.8	48.1	< .0001	-0.04	-0.18/ -0.19	0.10/ 0.12
Partnership (%)	43.1	65.3	83.7	89.9	< .0001	0.28	0.35/ 0.30	0.06/ 0.04
Children (%)	76.6	80.6	85.4	85.7	< .0001	0.08	0.07/ 0.06	0.03/ 0.03
Socioeconomic status	10.2	12.1	13.1	13.5	< .0001	0.16	0.12/ 0.09	0.10/ 0.09
Employment	45.5	57.9	64.0	58.4	< .05	0.03	0.03/ 0.01	0.02/ 0.01
Migrant	35.2	28.9	22.4	19.8	< .0001	-0.09	-0.06/ -0.04	-0.06/ -0.06
Smoker (%)	29.8	24.5	19.7	14.5	< .0001	-0.10	-0.06/ -0.06	-0.08/ -0.08
Obesity (BMI ≥ 30 kg/m^2^, %)	29.5	26.5	24.8	24.1	< .01	-0.02	0.02/ 0.06	-0.05/ -0.06
Alcohol (in gram/day, M, SD)	9.7 (20.5)	10.5 (17.1)	11.2 (16.5)	11.7 (17.0)	< .0001	0.03	0.06/ 0.07	-0.02/ -0.00
Hyperlipidemia (%)	41.9	37.0	34.4	35.0	< .0001	-0.03	0.03/ 0.04	-0.06/ -0.05
Diabetes ^1)^ (%)	14.0	10.7	8.6	8.3	< .0001	-0.05	-0.02/ -0.00	-0.05/ -0.04
CVD ^2)^ (%)	15.8	12.0	9.6	9.9	< .0001	-0.03	-0.00/ 0.01	-0.05/ -0.04
Depression (PHQ-9 ≥ 10, %)	24.5	18.3	6.3	2.8	< .0001	-0.32 ^4)^	-0.22/ -0.17	-0.25/ -0.22
Suicidal ideation (%)	25.7	16.5	6.3	3.4	< .0001	-0.20	-0.13/ -0.10	-0.17/ -0.14
Generalized anxiety (GAD ≥ 3, %)	19.1	13.4	5.9	2.9	< .0001	-0.22 ^4)^	-0.16/ -0.14	-0.17/ -0.14
Panic attack (past 4 weeks)	12.6	10.2	5.1	3.5	< .0001	-0.11	-0.09/ -0.07	-0.08/ -0.06
Social Phobia (Mini-Spin ≥ 6)	10.8	8.1	3.2	1.8	< .0001	-0.12	-0.08/ -0.06	-0.11/ -0.09
CDS-2 ≥ 3 (Depersonalisation)	5.9	1.9	0.5	0.2	< .0001	-0.11	-0.07/ -0.05	-0.08/ -0.06
Sleep disorder ^3)^ (%)	37.0	30.6	18.9	13.1	< .0001	-0.16	-0.12/ -0.09	-0.13/ -0.11
Type D Helmholtz	45.0	38.5	23.0	14.6	< .0001	-0.21	-0.11/ -0.09	-0.19/ -0.17
Analgetics	13.3	10.7	9.2	8.2	< .0001	-0.03	-0.03/ -0.03	-0.02/ -0.02
Opioids	3.6	1.8	1.3	1.2	< .0001	-0.03	-0.02/ -0.02	-0.03/ -0.03
Antidepressants	12.5	10.2	4.8	3.6	< .0001	-0.11	-0.10/ -0.08	-0.06/ -0.05
Physician visit last month	50.5	48.0	42.2	40.7	< .0001	-0.05	-0.05/ -0.04	-0.03/ -0.02
Overall health (M, SD)	6.4 (1.9)	6.7 (1.8)	7.3 (1.6)	7.7 (1.5)	< .0001	0.22	0.12/ 0.08	0.19/ 0.18

Note: Cochran-Armitage Test or Jonckheere-Terpstra Test were performed for group comparisons.

^1)^ based on medication, HbA1c, medical diagnosis

^2)^ CVD: Myocardial Infarction, CHD, Stroke, Peripheral Arterial Disease

^3)^ at least half the days/ everyday

^4)^ based on scale sum score

The majority reported high (55%), followed by very high (27%) support. Fifteen percent reported moderate and only 3% low support. Age did not play a role, however, women reported lower support than men. Participants with low support were at a clear social disadvantage regarding partnership, children, socioeconomic status, lack of employment, and a migration background. They had by far the highest rates of cardiovascular risk (smoking, obesity, hyperlipidemia), physical disease (cardiovascular, diabetes). They had the highest rates of distress (depression, suicidal ideation, GAD, panic attacks, social phobia, depersonalization, sleep disorder, Type D personality) and the highest intake of analgetics, opioids and antidepressants and most frequently visited physicians.

### Emotional-informational and tangible support as a predictor of mental disease

In multivariate analyses we predicted distress by the two subscales of social support. Even when we adjusted for age, sex, CVD, CVD risk, SES and number of persons in the household, both emotional-informational and tangible support were highly predictive of lower depression, suicidality, generalized anxiety, panic, social phobia, depersonalization, sleep disorder. For social anxiety and Type D personality, emotional support seems to be more important than practical support. When controlling for age, sex, CVD, CVD risk, SES, and number of persons in the household, for all listed mental problems, except for depersonalization, emotional-informational support appears to be a better predictor than tangible support. Overall, higher age and social status were associated with lower distress, whereas female sex and the presence of CVD were associated with higher distress. The results are displayed in Table 3.

**Table 3:** Independent predictors for distress symptoms in logistic regression models.

Distress	Odds Ratio*	95%-CI	Odds Ratio^†^	95%-CI
**Depression**				
Emotional-informational^‡^	0.66	(0.62–0.70)	0.71	(0.67–0.76)
Tangible^‡^	0.58	(0.54–0.61)	0.57	(0.54–0.61)
**General anxiety**				
Emotional-informational ^‡^	0.70	(0.66–0.74)	0.75	(0.70–0.80)
Tangible^‡^	0.66	(0.62–0.70	0.63	(0.59–0.67)
**Suicidality**				
Emotional-informational ^‡^	0.70	(0.66–0.74)	0.77	(0.73–0.82)
Tangible^‡^	0.59	(0.55–0.62)	0.59	(0.56–0.63)
**Panic**				
Emotional-informational ^‡^	0.74	(0.69–0.79)	0.79	(0.73–0.85)
Tangible^‡^	0.76	(0.71–0.81)	0.75	(0.69–0.80)
**Social anxiety**				
Emotional-informational ^‡^	0.75	(0.70–0.81)	0.81	(0.75–0.89)
Tangible^‡^	0.63	(0.58–0.68)	0.60	(0.56–0.66)
**Sleep disorder**				
Emotional-informational ^‡^	0.77	(0.74–0.80)	0.82	(0.79–0.86)
Tangible^‡^	0.74	(0.71–0.77)	0.73	(0.70–0.77)
**Depersonalization**				
Emotional-informational ^‡^	0.61	(0.52–0.70)	0.64	(0.53–0.76)
Tangible^‡^	0.52	(0.44–0.61)	0.49	(0.41–0.59)
**Type D Personality**				
Emotional-informational ^‡^	0.79	(0.76–0.83)	0.81	(0.78–0.85)
Tangible^‡^	0.65	(0.63–0.68)	0.65	(0.62–0.68)

* univariate analysis

† Adjusted for the following variables: age, sex, cardiovascular disease, cardiovascular risk factor, socioeconomic status, one-person household

‡ Calculated by explorative factor analysis

Abbreviations: CI = Confidence interval

## Discussion

The postulated hypotheses were borne out by analyzing two independent population-based subsamples. First, we successfully derived a brief scale with only six items (see S2 Table). We could reproduce the factor structure of previous brief scales with two independent dimensions, emotional-informational support and tangible support assessed by three items each. Second, confirmatory factor analysis showed a very good fit for the bi-factorial model in comparison to the one-factor solution renderings support for the need of differentiation of both facets of social support. As summarized by [21], most of the studies using the MOS Social Support Survey show different factor structures depending on the chosen factor analytical approach. However, our findings based on a German population-based sample clearly indicate a two-factor structure regardless of the used methodological approach.

Different associations of some socio-demographic and health-related variables with emotional-informational and tangible support were observed, corroborating the assumption of differentiating effects of both facets of social support. People in a partnership are more likely to experience emotional-information support, whereas the experience of tangible support seems not to be correlated with having a partner. Type D personality and overall health seem to have a slightly stronger association with practical support. From a psychological perspective, the knowledge about the divergence of emotional and practical pathways might be crucial to provide adequate support. The relevance of emotional and practical support probably differs depending on situational variables and interindividual differences.

Nevertheless, similar to previous studies low social support was found generally in socially disadvantaged participants [31, 32]. Emotional-informational and tangible support were in sum consistently predictive of a better mental health, even when adjusting of determinants of distress such as female sex, low SES, and the presence of CVD. For social anxiety and Type D personality, emotional-informational support seems to be crucial. When adjusting for age, sex, CVD, CVD risk, SES, and number of persons in the household, emotional support appears to be more important to predict mental health. The results emphasize the practical implication of distinguishing between the emotional and practical dimension of social support.

However, one limitation of the study is the age range of the sample beginning with 35 years. Due to the lack of adolescents and adults in their twenties or early thirties in our sample, the full range of age related aspects with regard to dimensions of social support have not been investigated in this study. Especially the impact of perceived social support on personality development and health in critical stages of life while reaching adulthood needs further research with sound study designs.

Taking into consideration the strict time constraints to which large scale data collection are subject, extensive assessment tools are usually not applicable for these kind of survey research. The efficiency of this brief scale was demonstrated by presenting validation testing with a number of health related variables. Findings with external criteria were in line with results of other MOS Social Support Surveys (e.g. [16, 17, 19]). The generalizability of the study results is high because of the underlying population-based sample. With the BS6, we provide a reliable and valid short scale that can be applied in social surveys due to its brevity to assess emotional and practical dimensions of social support.

## Supporting information

S1 Table Sample characteristics based on sociodemographic variables. (DOCX)

S2 Table Brief Social Support Scale (BS6). (DOCX)
