# Supplementary material for: Emotional and tangible social support in a German population-based sample: Development and validation of the Brief Social Support Scale (BS6)
Source: PLoS One. 2017 Oct 12;12(10):e0186516. doi: 10.1371/journal.pone.0186516 (PMC5638535; doi:10.1371/journal.pone.0186516)
Supplement: S1 Table — (DOCX) [file pone.0186516.s001.docx]

**S1 Table. Sample characteristics based on sociodemographic variables.**

|  | **Total sample (N=15,010)** |
| --- | --- |
|  | *Mean ± SD* |
| **Age** (years) | 54.9 ± 11.1 |
|  | *Percent (N)* |
| **Gender** (female) | 49.4 (7,426) |
| **Employment** (full-time or part-time) | 60.7 (9,136) |
| **Partnership** (yes) | 81.2 (12,182) |
| **Living alone** (yes) | 14.5 (2,137) |
| **Marital status** |  |
| married | 74.4 (11,160) |
| unmarried | 10.7 (1,609) |
| divorced | 8.6 (1,288) |
| widowed | 4.8 (712) |
| separated | 1.5 (228) |
| **Education** |  |
| less than 10^th^ grade | 39.0 (5,833) |
| completed 10^th^ grade | 23.0 (3,429) |
| completed high school | 36.8 (5,505) |
| other | 0.6 (83) |
| no graduation | 0.6 (93) |
