# Supplementary material for: Emotional and tangible social support in a German population-based sample: Development and validation of the Brief Social Support Scale (BS6)
Source: PLoS One. 2017 Oct 12;12(10):e0186516. doi: 10.1371/journal.pone.0186516 (PMC5638535; doi:10.1371/journal.pone.0186516)
Supplement: S2 Table — (DOCX) [file pone.0186516.s002.docx]

**S2 Table. Brief Social Support Scale (BS6).**

| **English** | **German** |
| --- | --- |
| If you needed it, how often is someone available… | Wie häufig steht Ihnen folgende Unterstützung durch andere Menschen zur Verfügung? |
| *Tangible support* | |
| To take you to the doctor if you need it | Jemand, der Sie zum Arzt fährt, wenn es nötig ist |
| To prepare your meals if you are unable to do it yourself | Jemand, der Ihnen Essen zubereitet, wenn Sie dazu nicht in der Lage sind |
| To help with daily chores if you were sick | Jemand, der Ihnen bei alltäglichen Arbeiten hilft, wenn Sie krank sind |
| *Emotional-informational support* | |
| To give you good advice about a crisis | Jemand, der Ihnen in schwierigen Situationen gute Ratschläge gibt |
| To confide in or talk to about yourself or your problems | Jemand, dem Sie sich anvertrauen oder mit dem Sie über persönliche Probleme sprechen können |
| Who understands your problems | Jemand, der Ihre Probleme versteht |
| *Rating options: 1= never, 2=occasionally, 3= mostly to 4 = always* | *Antwortoptionen: 1= nie, 2=manchmal, 3= meist bis 4 = immer* |
